# Relative validity of a web-based food frequency questionnaire for patients with type 1 and type 2 diabetes in Denmark

**DOI:** 10.1038/nutd.2016.40

**Published:** 2016-09-26

**Authors:** S M R Bentzen, V K Knudsen, T Christiensen, B Ewers

**Affiliations:** 1 Department of Nutrition and Food Service, Steno Diabetes Center, Gentofte, Denmark; 2 Division of Risk Assessment and Nutrition, National Food Institute, Technical University of Denmark, Søborg, Denmark

## Abstract

**Background:**

Diet has an important role in the management of diabetes. However, little is known about dietary intake in Danish diabetes patients. A food frequency questionnaire (FFQ) focusing on most relevant nutrients in diabetes including carbohydrates, dietary fibres and simple sugars was developed and validated.

**Objectives:**

To examine the relative validity of nutrients calculated by a web-based food frequency questionnaire for patients with diabetes.

**Design:**

The FFQ was validated against a 4-day pre-coded food diary (FD). Intakes of nutrients were calculated. Means of intake were compared and cross-classifications of individuals according to intake were performed. To assess the agreement between the two methods, Pearson and Spearman's correlation coefficients and weighted kappa coefficients were calculated.

**Subjects:**

Ninety patients (64 with type 1 diabetes and 26 with type 2 diabetes) accepted to participate in the study. Twenty-six were excluded from the final study population.

**Setting:**

64 volunteer diabetes patients at the Steno Diabetes Center.

**Results:**

Intakes of carbohydrates, simple sugars, dietary fibres and total energy were higher according to the FFQ compared with the FD. However, intakes of nutrients were grossly classified in the same or adjacent quartiles with an average of 82% of the selected nutrients when comparing the two methods. In general, moderate agreement between the two methods was found.

**Conclusion:**

The FFQ was validated for assessment of a range of nutrients. Comparing the intakes of selected nutrients (carbohydrates, dietary fibres and simple sugars), patients were classified correctly according to low and high intakes. The FFQ is a reliable dietary assessment tool to use in research and evaluation of patient education for patients with diabetes.

## Introduction

In Denmark, patients with diabetes are recommended to eat a healthy diet corresponding to the food-based dietary guidelines for the general Danish population. Both quantity and quality of carbohydrates influence blood glucose postprandial levels, with the total intake of carbohydrates being the primary predictor of glycemic response.^1^ Hence, in the dietary management of patients with diabetes, dieticians focus on assessing and educating patients in adjusting carbohydrate intakes, including amounts and types (for example, dietary fibres and simple sugars) of carbohydrates. Intake of carbohydrates derived from vegetables, fruits, whole grains, legumes and cereals is recommended over intake of carbohydrate from sources containing high amounts of fat, sugars or sodium. Patients with diabetes should consume at least the amount of dietary fibres and whole grains recommended for the general population in the Nordic countries.^2, 3, 4^

Among several methods to assess dietary intake, food frequency questionnaires (FFQs) are commonly used, since self-administered FFQs are less expensive and less time-consuming for participants and researchers compared with other dietary assessment methods such as 24-h recalls, food diaries or food records.^5^ A FFQ provides knowledge of the habitual dietary intake over a designated period of time.^6^

Several international studies have validated FFQs for the assessment of habitual dietary intakes in non-diabetic populations.^7, 8, 9^ Correspondingly, Danish FFQs have been developed and validated for different target populations over the years, with the most commonly used FFQ being from the early 1990s.^10, 11, 12^ This FFQ does not reflect today's food supply and intake of different carbohydrate-rich foods including high-fibre and high-sugar foods, which is most relevant in diabetes management.^10^ Currently, no Danish studies have been published investigating the validity of a FFQ targeting patients with diabetes, despite the importance of assessing and monitoring these patients' dietary intake over time in the management of diabetes.

The aim of the present study was to validate intake of nutrients focusing on carbohydrates, dietary fibres, simple sugars and total energy using a newly developed web-based FFQ and a 4-day food diary (FD) as reference.

## Subjects and methods

### Participants

The participants were from an out-patient clinic at Steno Diabetes Center (Gentofte, Denmark) based on the following inclusion and exclusion criteria. Inclusion criteria: >18 years of age and treated at Steno Diabetes Center. Exclusion criteria: mental illness in the form of dementia, psychosis, severe depression and severe (life-threatening) competitive disorder.

All patients gave written informed consent.

Twenty-three percent (*n*=90) of the 400 invited patients accepted to participate in the study. One patient was identified as mentally ill during the study and was excluded from the final study population. Furthermore, patients were excluded from the final study population if their registration in one of the two dietary assessments was not entirely completed or if only one of the two assessments (FFQ or FD) was completed (in a total of 25 patients), resulting in a final study population of 64 participants.

### Data collection

#### Food frequency questionnaire

An electronic, self-administered, semiquantitative FFQ was developed by dieticians and nutrition specialists at the Nutrition and Food Service Department at Steno Diabetes Center in collaboration with DTU National Food Institute, for diabetic patients. The FFQ consisted of 270 food items and mixed dishes. The portion sizes were estimated using household measures such as cups, glasses and tablespoon, or by means of a series of photographs from DANSDA (the Danish National Survey of Diet and Physical Activity)^13^ with varying sizes of commonly eaten food items or dishes high in carbohydrates, simple sugar or dietary fibres. The survey was implemented in LimeSurvey (San Francisco, CA, USA) and ran on a dedicated server provided by DTU National Food Institute.

To reflect changes in glycated haemoglobin (HbA1c), the FFQ was designed to cover intakes in the previous 3 months, and the question included the following answering categories: once or less per month, two to three times per month, one to two times per week, three to four times per week, five to six times per week, once each day, two to three times each day, four to five times each day, four or more times each day, six or more times each day, and, if never consumed, ‘none'. Additional questions concerning the frequency of different meals such as breakfast, lunch, dinner and snacks in the morning, afternoon and evening, respectively, were requested and frequencies of meals consumed away from home such as canteen, burger bar, restaurant and so on were included.

In advance of developing the FFQ, a small feasibility study was performed for final adjustment of questions in the FFQ.

Mean intaks of macro- and micronutrients recorded in FFQ were calculated using the software system General Intake Estimate System (GIES) developed at the National Food Institute (Mørkhøj, Denmark), including standard recipes and information on portion sizes from DANSDA. Nutritional data were obtained from the Danish Food Composition Database (www.foodcomp.dk).

#### Precoded food diary

The precoded FD used in the Danish National Survey of Dietary Habits^13^ was selected as the reference method in the present validation study, as this method has previously shown good validity of measuring the habitual dietary intake.^14^ In our validation study, the FD was completed for 4 consecutive days, 3 weekdays and 1 weekend day (Saturday or Sunday). The FD included precoded lines for the most commonly consumed foods and drinks in the Danish diet. It comprised breakfast, lunch, dinner and snacks in the morning, afternoon and evening, respectively, and for each meal the participants had the possibility of writing additional food items. Portion sizes were estimated using household measures such as cups, glasses and tablespoon or by means of a series of photographs with varying sizes of commonly eaten food items similar to those used for carbohydrate sources in the FFQ.

Mean intakes of macro- and micronutrients recorded in the precoded FD were calculated by using the same software system (GIES) as the FFQ, using the same recipes and food composition data.

#### Electronic medical records

Selected background variables (age, sex, body mass index (BMI), glycated haemoglobin, diabetes duration, type of diabetes and smoking habits) for the invited participants were extracted from electronic medical record at Steno Diabetes Center prior to the study.

### Statistical methods

All statistical analyses were performed in IBM Corp, SPSS Statistics for Windows, Version 20.0 (Armonk, NY, USA) For all statistical tests a significance level of *P*<0.05 were chosen. Normality of the dietary variables was assessed, and in cases of not normally distributed data, data were transformed. Data are presented as means and standard deviations (s.d.). Unpaired *t*-test was used for normally distributed continuous data and Mann-Whitney *U* test for non-normally distributedcontinuous data, in comparison of participants and non-respondents. Categorical data were compared using the chi-square test. Paired *t*-test was used for normally distributed data to compare mean intakes assessed by FFQ and FD. For normally distributed data Person's correlation coefficients were calculated and for non-normally distributed data Spearman's rank correlation was applied. Bland–Altman plot was used to identify extreme outliers and to identify agreement between the two dietary methods. Furthermore, individuals were divided into quartiles according to intake of macro- and micronutrients. Agreement between the two dietary methods was assessed using cross-classification; proportions of individuals who were categorised in the same or an adjacent quartile were assessed, and individuals in the opposite lowest/highest quartile were assessed and categorised as gross misclassification. Furthermore, kappa coefficients of agreement between the methods were estimated.

## Results

The participants in the present study were significantly younger and more patients had type 1 diabetes compared with non-responders. No other significant differences were observed between responders and non-responders in the invited population (Table 1).

In total, 90 patients accepted to participate in the study, of which 70% had type 1 diabetes and 30% had type 2 diabetes (Table 2). Patients with type 2 diabetes were significantly older, had a higher BMI and a shorter diabetes duration.

Comparison of intakes according to FFQ and FD showed a statistically significant difference in the reported energy intake for carbohydrates and dietary fibres, with a higher reported intake of both carbohydrates and dietary fibres according to the FFQ (Table 3). No differences were observed for total energy, protein, total fat, SFA (saturated fatty acid), MUFA (monounsaturated fatty acid), PUFA (polyunsturated fatty acid), simple sugars, alcohol, vitamin D, calcium, n-3 (omega-3 fatty acid) and n-6 (omega-6 fatty acid).

**Figure 1:**
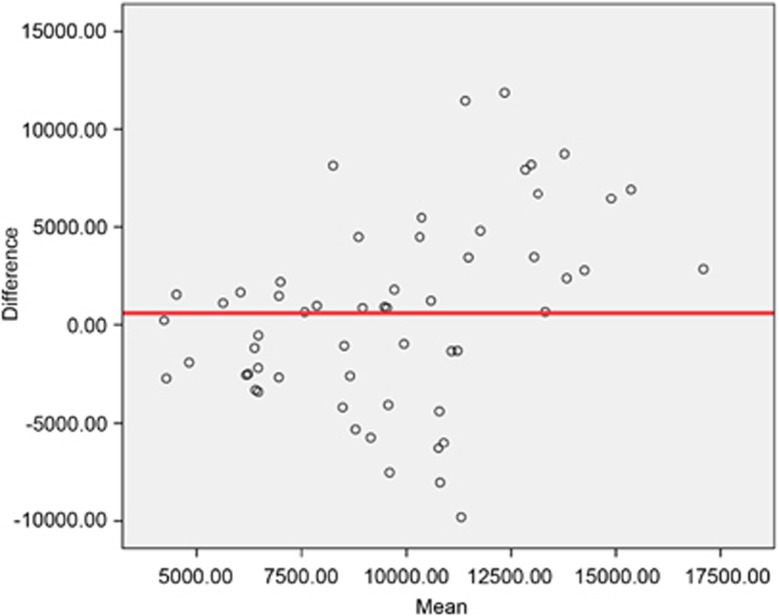
Bland-Altman plot for energy intake. Y-axis: difference in energy intakes according to FFQ and FD. X-axis: mean energy intake according to FFQ and FD.

Differences in energy intake according to FFQ and FD with Bland–Altman plot are shown in (Figure 1).

As shown in Table 4, correlation coefficients for the reported dietary intake according to FFQ are FD ranged from 0.30 to 0.70. Specifically, there was a high correlation between the FFQ and FD for total energy, carbohydrates, simple sugars and dietary fibres.

As shown in Table 5, the average percentage of individuals in the same or adjacent quartiles in the different variables of macro- and micronutrients intake was 79%, and ranged from 69.2 to 92.3%. The average percentage of gross misclassification was 4% and ranged from 1.5 to 7.7%. The weighted kappa coefficient ranged from −0.068 to 0.384.

## Discussion

To our knowledge, the present study is the first study to investigate the validity of a diabetes-specific FFQ. This FFQ, developed to assess dietary intake in patients with diabetes, showed good performance in ranking individuals with respect to high and low intake of selected nutrients, and to correctly classify individuals in quintiles of intakes compared with result from other studies validating FFQs.^10^

Generally, comparing mean intake of total energy and macro- and micronutrients between the two dietary assessment methods showed good consistency, although differences in intake of carbohydrates and dietary fibres were statistically significant, with considerably higher mean intake of carbohydrates and dietary fibres in the FFQ compared with the FD. The higher intake of carbohydrates and dietary fibres according to the FFQ could be due to overestimation by this method, possibly because of more detailed questions with focus on food items rich in carbohydrates and dietary fibres. It is known that excessively long lists of foods containing a certain nutrient can lead to overestimation of that nutrient.^15^ On the other hand the difference could also be due to an underestimation from the FD, possibly because of under-reporting, as the FD does not include as many specific high-fibre food items as the FFQ. In addition, subjects tend to under-report when completing food records, possibly because of the burden related to the registration or the awareness of their dietary intake resulting in altered dietary habits.^14^ However, the correlation coefficient and classification is of greater importance when investigating the relative validity.^14^ As mentioned previously, carbohydrates (including the type of carbohydrates) are important in the dietary management of diabetes to achieve a good metabolic control. Consequently, the correlations >0.50 found for carbohydrates, simple sugars and dietary fibres are highly valuable in our study, indicating that our FFQ is particularly capable of ranking individuals correctly in these essential nutrients. Correspondingly, when looking at cross-classification for carbohydrates and dietary fibres, individuals were correctly classified as having a high or a low intake with an average of 83% in the same or adjacent quartile and 84% in the same or adjacent quartile. Despite differences in absolute intake of carbohydrates and dietary fibres according to the two dietary assessment methods, the methods proved to be able to put individuals into the right classification, corresponding to the intention behind this newly developed FFQ.

Higher intakes in the FFQ compared with food records have been observed in validation studies by Brantsæter *et al.*^16^ and Rothenberg.^17^ In the study by Rothenberg, FFQ provided consistently higher intake of nutrients than food records and in the study by Brantsæter and colleagues the FFQ provided higher intake of the vast majority of nutrients, except for intake of total fat and iron.

In most validation studies, correlation coefficients between dietary assessment methods are considered poor if <0.30, fair if 0.30–0.49 and good if >0.50,^16^ demonstrating an overall fair consistency between the FFQ and the FD in our study, with average correlation coefficients at 0.44, ranging from 0.30 (MUFA) to 0.70 (alcohol) for daily intake of nutrients, which is in the same magnitude as found by Brantsæter *et al.*^16^ and Rothenberg.^17^

Total energy intake was one of the most important measures in our study, and a good correlation (0.50) was found. Furthermore, carbohydrates, simple sugars, dietary fibres and alcohol showed good correlations between the two methods. When allocating according to nutrient intake into quartiles for the two different methods and looking at cross-classification, individuals were generally correctly classified into the right quartile or the adjacent with an average of 79%, varying from 69 to 92%, thus indicating good agreement between the methods. Gross misclassification is averagely observed in 4%, varying from 1.5 to 7.7%. Classification into the same or adjacent quartile by the two dietary methods was similar to that reported in other validation studies.^18, 19, 20^ Hence the FFQ is an appropriate method for classification of individuals according to high or low intake of nutrients. Furthermore, the essential variables in relation to our study, total energy, carbohydrates, simple sugars and dietary fibres, are grossly correctly classified, with 80% being classified in the right or adjacent quartile, further underlining that our FFQ is highly capable of ranking participants according to intake of these important nutrients.

The Bland–Altman plot showed that the differences in energy intake between the two methods increased with higher mean energy intake. The same was observed in the validation study by Brantsæter *et al.*, where an increased intake of the vast majority of nutrients was associated with an increased difference between FFQ and FD in the respective study, indicating that our findings are common when comparing a FFQ with a prospective FD.^16^

The weighted kappa coefficients (*k*) ranged from −0.068 (total fat) to 0.384 (alcohol), indicating a poor (*k*<0.20) to fair (*k*=0.21–0.40) agreement, as earlier reported.^21^ This is in agreement with other findings.^8, 22^

FFQs have been shown to be an appropriate method for assessing diet in a wide variety of epidemiological settings. In comparison with short-term records, the FFQ provides a better approximation of the habitual diet over longer periods.^14^ However, there are errors associated with the use of all dietary assessment methods.

Several other issues in performing a validation should be considered:

First, in validation of dietary assessment methods, errors associated with the two methods should be independent.^20^ Among the feasible comparative methods available for validating a FFQ, food records are likely to have the smallest correlated errors.^14^ FFQ was selected as the dietary survey method since dietary intake could be assessed over a longer time period, it was less resource-intensive, and we expected it to be easier to administer with a web-based version. Thus, the response rate was expected to be much higher compared with the use of 4-7 days dietary recording, which is considered the most valid method within the dietary assessments.^23^ In the present study, we included a precoded 4-day FD method as reference method, since it is a validated dietary assessment method.^18^ Additionally, this reference method is minimally dependent on memory; thus it potentially eliminates recall bias detected in the FFQ, which is a strength in our study.

A potential limitation is the difference in the time frame between the two methods, with FFQ covering 3 months retrospective and FD only 4 days prospective. However, since the FFQ only covers diet in the last 3 months, and since our reference method (6 days) was collected in the same season, little seasonal variation is expected. Considering the specific nutrients of interest in our study, no appropriate biomarkers have yet been identified for carbohydrates, fibres and simple sugars, which were our nutrients of interest. Consequently, it was impossible to use more objective measures to examine specific intake of these nutrients.

The variability of different statistical methods used in our study could be both a strength and a weakness. The combination of different tests provides a good basis for the assessment of the relative validity, since there is no ‘gold standard' when it comes to selecting statistical methods in the validation process.^14^ At the same time, it could be a risk to use too many statistical tests when validating a dietary assessment method.

An additional strength in both dietary assessments was the implementation of photographs to help quantify portion sizes. Evidence indicates that the use of photographs improves the ability to register the true quantity of dietary intakes.^22^

The web-based FFQ ensured that questions regarding food items or dishes were answered with no possibility of skipping questions.

Finally, the study population represents a diabetes population. This is an important factor when validating a new dietary assessment method.^14^ Furthermore, the population was a representative sample of diabetic patients. The participants were slightly younger compared with non-participants, which was expected since participation in the study required computer and internet access, thus excluding some of the elder individuals invited into this study. Additionally, younger people tend to be more willing to participate in studies. Even if our participants seem to represent the target population, it is still likely that our participants are more aware of their dietary habits and intake, since they voluntarily agreed to participate. A larger population size would have been preferable. Gibson recommends an average of 100 participants in validation studies.^14^ We included 90 patients in the study, ending up with 64 participants in the final study population. This sample size may be sufficient since our study population is quiet homogenous in relation to dietary intake when further examined.

In our validation study, we included both type 1 and type 2 diabetes patients. This may have been a potential limitation as more type 1 diabetes patients compared with type 2 diabetes patients participated in our study. Type 1 diabetes patients are generally considered more skilled in carbohydrate counting, including portion size estimation and knowledge of food items such as carbohydrates, which could have increased the validity of our FFQ. However, this was not a limitation since our included participants with type 1 diabetes only had a limited knowledge of carbohydrate counting at the time of the data collection. Additionally, all our included participants were untrained in dietary assessment, and received the same instructions for filling in the dietary assessment methods initially.

## Conclusion

In conclusion, the web-based FFQ developed to assess diet in diabetes patients proved to be highly appropriate in ranking individuals according to high and low intake of nutrients, and thus proves to be a usable tool for future research and evaluation of dietary intakes in patients with type 1 and type 2 diabetes.

## Figures and Tables

**Table 1:** Characteristics of participants and non-respondents in the validation study

*Variables*	*Participants (* n = *90)*		*Non-respondents (* n = *319)*		P*-value*
	*Mean/*n	*±s.d.*	*Mean/*n	*±s.d.*	
Age (years)	50.4	16.7	55.8	16.6	0.010^a^
Sex (male/female)	55/36	60/40	166/153	52/48	0.156
BMI (kg m^−2^)	26.8	5.3	27.6	6.9	0.151^a^
HbA1C (mmol mol^−1^)	58.9	12.1	63.4	15.5	0.100^b^
Diabetes duration (years)	17.9	13.4	20.9	13.6	0.263^c^

	*Mean/*n	*%*	*Mean/*n	*%*	P*-value*
Insulin (no/yes)	8/82	9/91	37/282	12/88	0.450
Insulin pump (no/yes)	75/15	84/16	289/30	91/9	0.057
Diagnose type 1/type 2	64/26	70/29	178/130	56/41	0.036

Abbreviations: BMI, Body mass index; HbA1c, Glycated haemoglobin.

a *P*-values for difference are found with unpaired *t*-test for normal distributed continuous variables and by non-parametric test for non-normal distributed variables. *P*-values for categorical variables were calculated with chi-square test. *P*-values <0.05 are considered statistically significant.

b Logarithmic transformed data for calculating the *P*-value

c Square root transformed data for calculating the *P*-value.

**Table 2:** Demographic information on of the participants

*Variables*	*Type 1 (* n = *64)*		*Type 2 (* n = *26)*	
	*Mean*	*±s.d.*	*Mean*	*±s.d.*
Age (years) (range 17–80)	45.3	16.3	63.6	9.2
BMI (kg m^−1^)	24.9	3.7	31.4	5.7
HbA1c (mmol mol^−1^)	57.0	10.9	62.2	12.6
Diabetes duration (years)	19.9	15.0	13.0	6.6
	n	*%*	n	*%*
Sex (male/female)	40/24	63/37	14/12	54/46

Abbreviations: BMI, Body mass index; HbA1c, Glycated haemoglobin; T1DM, type 1 diabetes mellitus; T2DM, type 2 diabetes mellitus.

*P*-values for difference are found with unpaired *t*-test for normal distributed continuous variables and by non-parametric test for non-normal distributed variables. *P*-values for categorical variables were calculated with chi-square test. *P*-values <0.05 are considered statistically significant.

Logarithmic transformed data for calculating the *P*-value.

Square root transformed data for calculating the *P*-value.

**Table 3:** Estimated daily nutrient intake according to FFQ and FD

*Variables*	*FFQ (* n = *64)*			*FD (* n = *64)*			*Mean Diff.*	P*-value*
	*Mean*	*±s.d.*	*E%*	*Mean*	*±s.d.*	*E%*		
Energy (kJ per day)	11988	6766		9794	3263		2193	0.079^a^
Protein (g per day)	101	61	14	97	33	17	4	0.205^a^
Total fat (g per day)	99	59	31	93	39	36	5	0.780^a^
SFA (g per day)	34	21	11	35	17	13	2	0.117^a^
MUFA (g per day)	38	25	12	35	15	13	3	0.669^a^
PUFA (g per day)	18	13	6	14	6	5	4	0.087^a^
Carbohydrates (g per day)	362	245	51	239	83	41	123	**0.001**^a^
Simple sugar (g per day)	35	50		32	28		3	0.113^b^
Dietary fibre (g per day)	38	30		24	9		14	**0.000**^a^
Alcohol (g per day)	13	22	4	18	20	6	5	0.183^b^
Vit. D (μg per day)	3.7	2.7		5.1	6.4		1.4	0.195^a^
Ca (mg per day)	1564.8	1047.2		1248.2	454.7		316.6	0.213^a^
n-3 (g per day)	3.8	2.8		3.1	1.7		0.7	0.119^a^
n-6(g per day)	14.2	10.1		11.1	4.6		3.1	0.091^a^

Abbreviations: Ca, calcium; MUFA, monounsaturated fatty acids;n-3, omega-3 fatty acid; n-6, omega-6 fatty acid; PUFA, polyunsaturated fatty acids; SFA, saturated fatty acids; Vit. D, vitamin D;

E%, percentage of energy intake.

a Logarithmic transformed data for calculating the *P*-value.

b *P*-values for difference are found with unpaired *t*-test for normal distributed continuous variables and by non-parametric test for non-normal distributed variables. *P*-values for categorical variables were calculated with chi-square test. *P*-values <0.05 are considered statistically significant.

b Square root transformed data for calculating the *P*-value.

**Table 4:** Correlation coefficients of participants' intakes of macro and micronutrients according to FFQ and FD

*Variables*	*r*^a^	*Sig. (2-tailed)*
Energy	0.50	0.000^b^
Protein	0.49	0.000^b^
Total fat	0.36	0.003^b^
SFA	0.38	0.002^b^
MUFA	0.30	0.013^b^
PUFA	0.38	0.002^b^
Carbohydrates	0.51	0.000^b^
Simple sugars	0.53	0.000^c^
Fibres	0.50	0.000^b^
Alcohol	0.70	0.000^c^
Vit. D	0.37	0.003^b^
Ca	0.45	0.000^b^
n-3	0.36	0.004^b^
n-6	0.40	0.001^b^

Abbreviations: Ca, calcium; MUFA, monounsaturated fatty acids; n-3, omega-3 fatty acid; n-6, omega-6 fatty acid; PUFA, polyunsaturated fatty acids; SFA, saturated fatty acids; Vit. D, vitamin D; r, correlation.

a *r* >0.30, good relationship; *r* = 0.5–0.8, really good relationship; *r* >0.8 good.

b Spearman's correlation test for non-normally distributed data.

c Pearson's correlation test.

**Table 5:** Weighted kappa coefficients and classification of agreement between quartiles of macro- and micronutrients intake according to FFQ and FD

*Variance*	*Quartiles of nutrient intake per day - FFQ*	*Quartiles of nutrient intake per day - FD*	*In same or adjacent quartiles*	*Gross misclassi-fication*	*Weighted kappa*
	*Q (intake) (* n*)*	*Q (intake) (* n*)*	*(%)*	*(%)*	*k*
Energy (kJ per day)	**1** (<6330.3) *(17)* **2** (6303.4–9980.7) *(16)* **3** (9980.8–16941.4) *(17)* **4** (>16941.5) *(15)*	**1** (<7412.0) *(17)* **2** (7412.1–9404.7) *(17)* **3** (9404.8–12318.1) *(17)* **4** (>12318.2) *(14)*	80.1	4.6	0.18
Protein (g per day)	**1** (<50.0) *(16)* **2** (50.1–80.0) *(17)* **3** (80.1–136.8) *(17)* **4** (>136.9) *(15)*	**1** (<70.8) *(17)* **2** (70.9–94.8) *(17)* **3** (94.9–117.7) *(17)* **4** (>117.8) *(14)*	84.6	4.6	0.22
Total fat (g per day)	**1** (<55.5) *(16)* **2** (55.6–79.9) *(17)* **3** (80.0–135.2) *(17)* **4** (>135.3) *(15)*	**1** (<65.9) *(17)* **2** (66.0–87.7) *(17)* **3** (87.8–112.0) *(17)* **4** (>112.1) *(14)*	69.2	1.5	−0.07
SFA (g per day)	**1** (<18.4) *(16)* **2** (18.5–26.5) *(17)* **3** (26.6–44.4) *(17)* **4** (>44.5) *(15)*	**1** (<23.5) *(17)* **2** (23.6–33.7) *(17)* **3** (33.8–41.0) *(17)* **4** (>41.1) *(14)*	73.8	1.5	0.04
MUFA (g per day)	**1** (<19.9) *(16)* **2** (20.0–29.2) *(17)* **3** (29.3–50.2) *(16)* **4** (> 50.4) *(16)*	**1** (<25.3) *(16)* **2** (25.4–32.2) *(17)* **3** (32.3–42.6) *(16)* **4** (>42.7) *(16)*	70.8	3.0	0.04
PUFA (g per day)	**1** (<9.9) *(16)* **2** (10.0–13.7) *(17)* **3** (13.8–22.5) *(16)* **4** (>22.6) *(16)*	**1** (<10.1) *(16)* **2** (10.2–13.4) *(17)* **3** (13.5–17.9) *(16)* **4** (>18.0) *(16)*	75.4	4.6	0.20
Carbohydrates (g per day)	**1** (<171.8) *(16)* **2** (171.9–281.7) *(17)* **3** (281.8–564.9) *(16)* **4** (>564.0) *(16)*	**1** (<176.3) *(16)* **2** (176.4–234.3) *(17)* **3** (234.4–286.7) *(16)* **4** (>286.8) *(16)*	83.0	3.1	0.16
Simple sugar (g/day)	**1** (<9.8) *(16)* **2** (9.9–15.4) *(17)* **3** (15.4–30.5) *(16)* **4** (>30.6) *(16)*	**1** (<12.6) *(16)* **2** (12.7–24.4) *(17)* **3** (24.5–42.5) *(16)* **4** (>42.6) *(16)*	80.1	3.0	0.08
Dietary fibre (g per day)	**1** (<19.2) *(16)* **2** (19.3–28.2) *(17)* **3** (28.3–47.6) *(16)* **4** (>47.7) *(16)*	**1** (<16.9) *(16)* **2** (17.0–22.7) *(17)* **3** (22.8–31.2) *(16)* **4** (>31.3) *(16)*	83.6	6.2	0.18
Alcohol (g per day)	**1** (<1.9) *(16)* **2** (2.0–5.4) *(17)* **3** (5.4–12.1) *(16)* **4** (>12.1) *(16)*	**1** (<0.4) *(16)* **2** (0.5–12.2) *(17)* **3** (12.3–29.0) *(16)* **4** (>29.1) *(16)*	92.3	1.5	0.38
Vit. D (μg per day)	**1** (<2.0.) *(16)* **2** (2.1–3.1) *(17)* **3** (3.2–4.5) *(16)* **4** (>4.6) *(16)*	**1** (<2.1) *(16)* **2** (2.2–3–3) *(17)* **3** (3.4–5.5) *(16)* **4** (>5.6) *(16)*	70.7	6.2	0.20
Ca (mg per day)	**1** (<732.0) *(16)* **2** (732.1–1239.6) *(17)* **3** (1239.7–2110.3) *(16)* **4** (>2110.4) *(16)*	**1** (<869.1) *(16)* **2** (869.2–1255.2) *(17)* **3** (1255.3–1498.8) *(16)* **4** (>1498.9) *(16)*	78.4	4.6	0.16
n-3 (g per day)	**1** (<1.9) *(16)* **2** (2.0–2.8) *(17)* **3** (2.9–4.9) *(16)* **4** (>5.0) *(16)*	**1** (<1.9) *(16)* **2** (2.0–2.8) *(17)* **3** (2.9–3.7) *(16)* **4** (>3.8) *(16)*	80.0	7.7	0.06
n-6 (g per day)	**1** (<7.4) *(16)* **2** (7.5–10.5) *(17)* **3** (10.6–17.8) *(16)* **4** (>17.9) *(16)*	**1** (<7.7) *(16)* **2** (7.8–10.3) *(17)* **3** (10.4–13.7) *(16)* **4** (>13.8) *(16)*	75.5	3.0	0.18

Abbreviations: Ca, calcium; k, kappa; MUFA, monounsaturated fatty acids; n-3, omega-3 fatty acid; n-6, omega-6 fatty acid; PUFA, polyunsaturated fatty acids; SFA, saturated fatty acids; Vit. D, vitamin D.
